# Multidimensional biomarker predicts disease control in response to immunotherapy in recurrent or metastatic head and neck squamous-cell carcinoma

**DOI:** 10.1007/s00432-023-05205-z

**Published:** 2023-08-08

**Authors:** Kevin C. Flanagan, Jon Earls, Ian Schillebeeckx, Jeffrey Hiken, Rachel L. Wellinghoff, Natalie A. LaFranzo, Zachary S. Bradley, Joey Babbitt, William H. Westra, Raymond Hsu, Lincoln Nadauld, Howard Mcleod, Sean D. Firth, Brittany Sharp, Josh Fuller, Vera Vavinskaya, Leisa Sutton, Ida Deichaite, Samuel D. Bailey, Vlad C. Sandulache, Matthew J. Rendo, Orlan K. Macdonald, Karim Welaya, James L. Wade, Andrew W. Pippas, Jennifer Slim, Bruce Bank, Steven J. Saccaro, Xingwei Sui, Adil Akhtar, Savitha Balaraman, Steven E. Kossman, Scott A. Sonnier, Todd D. Shenkenberg, Warren L. Alexander, Katherine A. Price, Charles L. Bane, Jessica Ley, David N. Messina, Jarret I. Glasscock, Ezra E. W. Cohen, Douglas R. Adkins, Eric J. Duncavage

**Affiliations:** 1 Cofactor Genomics, Inc., 4044 Clayton Ave, St. Louis, MO 63110 USA; 2 Department of Pathology, Icahn School of Medicine at Mount Sinai, New York, NY USA; 3 Vision Radiology, Dallas, TX USA; 4 Intermountain Healthcare, St. George, UT USA; 5 Division of Hematology and Oncology, UCSD Moores Cancer Center, La Jolla, CA USA; 6 Appalachian Regional Healthcare, Hazard, KY USA; 7 Bobby R. Alford Department of Otolaryngology–Head and Neck Surgery, Baylor College of Medicine, Houston, TX USA; 8 Hematology and Oncology, Brooke Army Medical Center, San Antonio, TX USA; 9 Cancer Care Northwest, Spokane Valley, WA USA; 10 CoxHealth Medical Oncology, Springfield, MO USA; 11 Decatur Memorial Hospital, Decatur, IL USA; 12 John B Amos Cancer Center, Columbus Regional Research Institute, Centricity Research, Columbus, GA USA; 13 Multicare Institute for Research and Innovation, Tacoma, WA USA; 14 Northwest Oncology and Hematology, Elk Grove Village, IL USA; 15 Ochsner Lafayette General Medical Center, Lafayette, LA USA; 16 Providence Regional Cancer System, Lacey, WA USA; 17 Revive Research Institute, Sterling Heights, MI USA; 18 Sharp Clinical Oncology Research, San Diego, CA USA; 19 Touro Infirmary, New Orleans, LA USA; 20 Valley Cancer Associates, Harlingen, TX USA; 21 William Beaumont Army Medical Center and The Geneva Foundation, Fort Bliss, TX USA; 22 Mayo Clinic, Rochester, MN USA; 23 Dayton Physicians Network/Precision Cancer Research, Dayton, OH USA; 24 Division of Oncology, Department of Medicine, Washington University School of Medicine, St. Louis, MO USA; 25 Department of Pathology and Immunology, Washington University School of Medicine, St. Louis, MO USA

**Keywords:** HNSCC, Biomarker, Immune checkpoint inhibitors, PD-L1, PD-1, Pembrolizumab

## Abstract

**Purpose:**

Anti-PD-1 therapy provides clinical benefit in 40–50% of patients with relapsed and/or metastatic head and neck squamous cell carcinoma (RM-HNSCC). Selection of anti- PD-1 therapy is typically based on patient PD-L1 immunohistochemistry (IHC) which has low specificity for predicting disease control. Therefore, there is a critical need for a clinical biomarker that will predict clinical benefit to anti-PD-1 treatment with high specificity.

**Methods:**

Clinical treatment and outcomes data for 103 RM-HNSCC patients were paired with RNA-sequencing data from formalin-fixed patient samples. Using logistic regression methods, we developed a novel biomarker classifier based on expression patterns in the tumor immune microenvironment to predict disease control with monotherapy PD-1 inhibitors (pembrolizumab and nivolumab). The performance of the biomarker was internally validated using out-of-bag methods.

**Results:**

The biomarker significantly predicted disease control (65% in predicted non-progressors vs. 17% in predicted progressors, p < 0.001) and was significantly correlated with overall survival (OS; p = 0.004). In addition, the biomarker outperformed PD-L1 IHC across numerous metrics including sensitivity (0.79 vs 0.64, respectively; p = 0.005) and specificity (0.70 vs 0.61, respectively; p = 0.009).

**Conclusion:**

This novel assay uses tumor immune microenvironment expression data to predict disease control and OS with high sensitivity and specificity in patients with RM-HNSCC treated with anti-PD-1 monotherapy.

**Supplementary Information:**

The online version contains supplementary material available at 10.1007/s00432-023-05205-z.

## Background

Head and Neck Cancer (HNC) is the seventh most common cancer worldwide with 890,000 new cases and 450,000 deaths per year (Pulte and Brenner 2010; Chow 2020). Despite advances in diagnosis and treatment in the curative setting, recurrent or metastatic disease (or both) develops in more than 65% of patients with squamous cell cancer of the head and neck (HNSCC; Argiris et al. 2008).

Based on a 2008 clinical trial, the standard of care for recurrent and metastatic squamous cell carcinoma of the head and neck (RM-HNSCC) was platinum-based chemotherapy along with 5-fluorouracil and the EGFR inhibitor cetuximab (EXTREME regimen; Vermorken et al. 2008; Sacco and Cohen 2015; Le et al. 2020). In 2016, the anti-PD-1 therapies pembrolizumab and nivolumab were approved for the treatment of platinum-pretreated RM-HNSCC, providing meaningful improvement in the standard of care for a subset of patients (Larkins et al. 2017; Ferris et al. 2016; Muro et al. 2016). In 2019, approval was extended to pembrolizumab as first-line treatment both as a monotherapy and in combination with platinum-based chemotherapy (combination therapy; Cohen et al. 2019a). Today, the standard of care for most patients is first line anti-PD-1 therapy for RM-HNSCC and second line anti-PD-1 therapy for platinum-pretreated RM-HNSCC (Pfister et al. 2023). Unfortunately, only 13–25% of patients’ tumors respond to anti-PD-1 monotherapy and 36% of patients respond to combination therapy (Le et al. 2020). Identifying those patients who will benefit from anti- PD-1 therapy has been an important goal, primarily utilizing the on-label companion diagnostic (CDx), a PD-L1 immunohistochemistry (IHC) assay.

PD-L1 IHC is a widely used biomarker for predicting clinical benefit in response to PD-1 inhibitors across many cancer types. Unfortunately, PD-L1 IHC testing in HNSCC, as in other cancer types, has relatively high sensitivity but poor specificity for predicting response and disease control (poor positive predictive value, PPV; Lu et al. 2019; Burtness et al. 2019; Brockstein and Vokes 2023). KEYNOTE-048 investigated whether pembrolizumab, as a monotherapy or in combination with chemotherapy, improved overall survival (OS) compared with cetuximab plus chemotherapy in participants with previously untreated RM-HNSCC (Burtness et al. 2019). In this study, PD- L1 IHC was used with combined positive score (CPS) thresholds of CPS ≥ 20 and CPS ≥ 1. Monotherapy anti-PD-1 patients with CPS ≥ 20 had a 53% Disease Control Rate (DCR), and patients with CPS ≥ 1 had a 47% DCR, compared to a 44% DCR in the total patient population, regardless of CPS. Thus, PD-L1 IHC had only a marginal benefit in predicting clinical benefit for pembrolizumab monotherapy.

Because current biomarkers like PD-L1 IHC have a low PPV, clinicians are increasingly using combination therapy as the default treatment (Le et al. 2020). In KEYNOTE-048, the combination chemotherapy plus pembrolizumab cohort had a DCR of 64%, higher than pembrolizumab monotherapy regardless of PD-L1 status (Burtness et al. 2019). However, the risk of grade 3–5 adverse events is far higher with combination therapy or EXTREME when compared to monotherapy (Cohen et al. 2019b; Burtness et al. 2019). Therefore, to improve patient outcomes and better manage toxicity risks, it is important that a diagnostic accurately identify the subset of patients who will respond to monotherapy.

Because of the negative impact on patients and the increased healthcare burden, there is a strong need for more robust and effective methods of predicting disease control in response to PD-1 inhibitors. Building on our previous work (LaFranzo et al. 2020; Schillebeeckx et al. 2020, 2022), we developed an RNA-sequencing-based classifier called OncoPrism^®^-HNSCC to predict disease control with increased sensitivity and specificity compared to PD-L1 IHC in patients with RM-HNSCC treated with anti-PD-1 monotherapy. OncoPrism-HNSCC requires as little as two 10 μm formalin-fixed, paraffin-embedded HNSCC tumor tissue sections and classifies patients according to their likelihood of progressing on anti-PD-1 monotherapy with high sensitivity and specificity.

## Methods

### Study design and participants

Patients were recruited from eleven study sites across the USA: Washington University in St. Louis (St. Louis, MO), University of California San Diego (San Diego, CA), MultiCare Institute for Research and Innovation (Tacoma, WA), Ochsner Lafayette General Medical Center (Lafayette, LA), Dayton Physicians Network (Dayton, OH), Intermountain Healthcare (Salt Lake City, UT), Brooke Army Medical Center (Fort Sam Houston, Texas), Baylor College of Medicine (Houston, TX), Cancer Care Northwest (Spokane, WA), Mayo Clinic (Rochester, MN), and Northwest Oncology and Hematology (Hoffman Estates, IL).

Patients were enrolled in the retrospective, observational study following the inclusion and exclusion criteria outlined below. Eligible patients had recurrent or metastatic histologically or cytologically confirmed HNSCC and were treated with anti-PD-1 monotherapy as the first-line treatment for their recurrent or metastatic disease. Tissue analyzed in the study was from tumors biopsied prior to treatment and was formalin-fixed and paraffin embedded using standard protocols. De-identified, FFPE pre-treatment tumor biopsy specimens were provided to Cofactor Genomics for OncoPrism-HNSCC and PD-L1 IHC analysis. The 10% of samples with the longest time between biopsy and treatment (> 22.4 months) were removed from the study. Following anti-PD-1 treatment, patients must have had clinician-evaluated tumor response to immunotherapy using clinical assessment (RECIST, PERCIST, or other clinical criteria as appropriate in standard of care) to calculate disease control rate (DCR). Exclusion criteria included immunotherapy received in combination with any other therapy modality including radiation therapy, platinum-based chemotherapy, or taxane. Patients with insufficient tissue for analysis were also excluded from the study. Primary or metastatic tumor specimens were accepted, but metastatic tumors from bone or liver were not included.

The study protocol, “A Multicenter Cancer Biospecimen Collection Study” is registered as “NCT04510129—Predicting Immunotherapy Efficacy From Analysis of Pre-treatment Tumor Biopsies (PREDAPT)” on clinicaltrials.gov. The study protocol was approved by institutional review boards at either the study (WCG IRB) or site level, as appropriate. All patients provided signed, informed consent to participate, or consent was waived for deceased patients according to study protocol. Independent data monitoring was conducted by the study clinical research organization Curebase, Inc (San Francisco, CA).

### RNA extraction

RNA was extracted using RNAstorm (Cell Data Sciences, Fremont, CA) according to the manufacturer’s instructions. RNA quantity was assessed by the High Sensitivity RNA Qubit assay (Thermo Fisher Scientific, Waltham, MA). A predefined yield of 40 ng FFPE RNA was used as the minimum QC threshold. Quality of the RNA was assessed using a bioanalyzer (Agilent Technologies, Santa Clara, CA), and a DV200 of 20% or greater was used as the minimum threshold.

### Library preparation and sequencing

Libraries were prepared using the QuantSeq 3’ mRNA-Seq Library Prep Kit FWD for Illumina (Lexogen, Inc., Greenland, NH), following the manufacturer’s instructions with the protocol alterations noted as follows. RNA input into library preparation was 40 ng for all samples. UMI Second Strand Synthesis Module for QuantSeq FWD (Lexogen, Inc., Greenland, NH) replaced Second Strand Synthesis Mix 1 in the workflow. All samples were processed with an OncoPrism-HNSCC Positive Control, OncoPrism-HNSCC Negative Control, and a No Template Control. The Positive (high scoring) and Negative (low scoring) controls were RNA extracted from RM-HNSCC samples as described above. Final libraries were sequenced to a minimum depth of 10 million single-end 75 base pair reads on a NextSeq500 (Illumina, San Diego, CA), following the manufacturer’s protocols.

### Immunohistochemistry

PD-L1 staining was performed by Mosaic Labs (Lake Forest, CA) using the 22C3 pharmDx antibody (Agilent Technologies, Inc., Santa Clara, CA) or NeoGenomics Laboratories (Fort Myers, FL) using the PD-L1 22C3 FDA (KEYTRUDA^®^) for HNSCC Head and Neck stain. CPS assessment was performed by W.H.W. or by NeoGenomics. H&E staining was performed by NeoGenomics as part of the PD-L1 22C3 test or at Cofactor Genomics using xylene substitute Slide Brite (Newcomer Supply, Middleton, WI), as detailed by manufacturers. Samples were assessed for tumor purity by a board- certified pathologist (EJD), with a minimum 10% tumor cellularity required for inclusion.

### Processing of RNA sequencing data

FASTQ files were preprocessed with trim_galore/cutadapt version 0.4.1 to remove adapter sequences as well as reads with PHRED quality scores < 20 and reads that were < 20 bp. The trimmed reads were aligned to the human genome GRCh38 with STAR version 2.5.2a using the two-pass method as previously described (Schillebeeckx et al. 2020). Read counts were generated using htseq-count version 0.9.1 and annotation from Gencode version 22 (Schillebeeckx et al. 2020). Only samples with a minimum of 30% exonic alignment and at least 800,000 unique deduplicated counts were included in the study.

### Feature selection and biomarker training

**Fig. 1:**
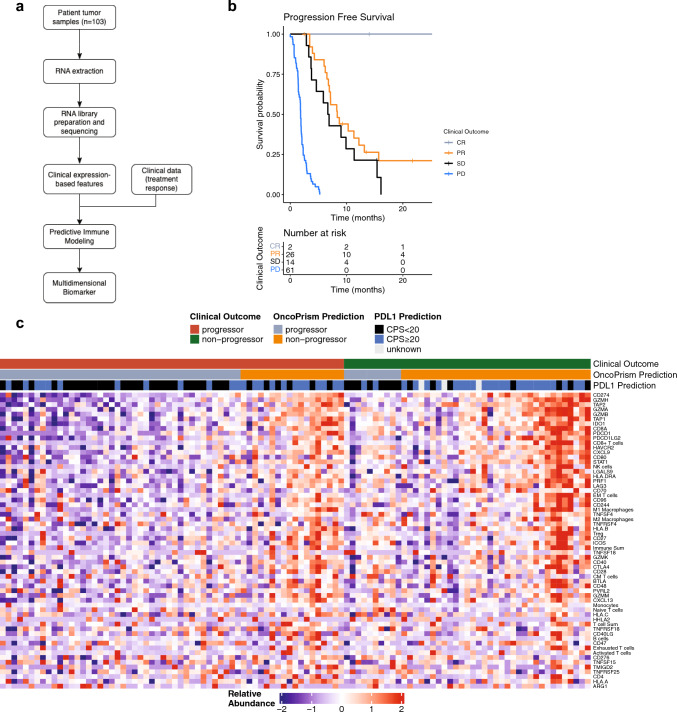
RNA-seq data was used to build the biomarker. **A** A multidimensional biomarker was trained using tumor specimen RNA-seq gene expression data and clinical response data from 103 HNSCC patients. Logistic regression and forward feature selection were used to integrate the data (“Predictive Immune Modeling”) and produce a multidimensional biomarker. **B** Patients were segregated by outcome and evaluated for progression free survival (PFS). CR, complete response; PR, partial response; SD, stable disease, PD, progressive disease. CR, PR, and SD together constitute “non-progressors”. PD patients are “progressors”. **C** Candidate feature relative abundance is shown for each patient sample. Features are gene expression data or gene signatures associated with specific immune cell types. Patient samples are sorted by outcome label (true outcome), then by OncoPrism Score and PD-L1 IHC prediction

103 patients were used to train the OncoPrism-HNSCC biomarker. As described previously, forward feature selection was performed with a logistic regression on 62 immunomodulatory features to generate candidate biomarkers (Fig. 1C and Table S1; Schillebeeckx et al. 2020, 2022). Log2 values between 10^–3^ and 10^2^ for the regularization parameter C were considered.

Candidate biomarkers were considered for their analytical reproducibility (variance) and clinical performance (area under the curve, AUC), and the final model was chosen to balance the two. Briefly, nine replicates from 6 patients (54 samples) were used to measure the variance of scores of candidate biomarkers. OncoPrism Scores for each replicate in the analytic variability dataset were generated and mean centered for each biological sample. A single measure of variability for each candidate model, defined as 4 times the standard deviation of the mean centered OncoPrism Scores (4*std), was used to evaluate variance. The final model was chosen to maximize clinical performance (AUC ≥ 0.76) while minimizing analytical variability (4*std ≤ 20).

Training and cross validation was performed using observational FFPE samples from patients treated with monotherapy pembrolizumab or nivolumab. The clinical cut-offs for the test system were established using the two endpoints identified for the clinical study: clinical outcome (CR, PR, SD, and PD, defined by RECIST criteria; Eisenhauer et al. 2009) and OS (median months survived from the start of therapy to death or last contact). CR, PR, and SD patients were considered “non-progressors” while PD patients were considered “progressors”.

Using a bootstrapping approach, scores were generated for each patient to evaluate biomarker performance and choose a threshold. Considering *n* unique patient samples, patients are chosen *n* times with replacement and used for training a model. Using this trained model, a score (“out-of-bag score”) is generated for the remainder patients. This process was done 1000 times, and the out-of-bag score of each patient was averaged to generate the final OncoPrism Score. This method minimizes bias and therefore best approximates future performance in an independent patient cohort.

### OncoPrism scores and prediction

The OncoPrism-HNSCC biomarker generates an OncoPrism Score from 0 to 100 that correlates with disease control and OS of HNSCC patients treated with anti-PD-1 monotherapy treatment. Higher OncoPrism Scores represent higher confidence by the model that the patient will be a non-progressor. The median score (46) was used to set the threshold between predicted progressor (scores less than the median) and predicted non-progressors (scores greater than the median).

### Statistics

Statistical analysis was performed using R (R Core Team 2021). Heatmap was made using the “ComplexHeatMap” and “circlize” packages (Gu et al. 2014, 2016). Survival figures and analysis were done using the “survminer” and “survival” packages, and significance was determined using log rank methods (Therneau and Grambsch 2000; Therneau 2023). DCR across quartiles, biomarker predictions and other groups, overall response rate (ORR), sensitivity, and specificity were compared using Fisher’s exact test.

## Results

### Patient selection

Pre-treatment FFPE tumor samples were collected from the 172 patients with RM-HNSCC enrolled under the internal review board-approved PREDAPT protocol. Thirty patients were removed from the study for pre-analytical reasons such as incomplete data or unacceptable tissue type. All remaining patients received at least one dose of anti-PD-1 monotherapy (following sample collection) and had documented anti-PD-1 outcome data. Following tumor cellularity estimation, RNA extraction, library preparation, and sequencing, 103 samples from 11 clinical sites met all inclusion/exclusion and quality control criteria (Table S2). Table 1 compares the patient population for this study (“OncoPrism”) with two other large ICI studies in RM-HNSCC (Hanna et al. 2018; Burtness et al. 2019).

**Table 1:** Patient data (n=103)

Characteristic		Burtness et al.	Hanna et al.	OncoPrism
Patient number	n	301	126	103
Age (median)	Age	62	57	67
Gender	Male	83%	83%	81%
	Female	17%	17%	18%
Smoking status	Current or Former	79%	52%	72%
	Never	21%	48%	19%
ECOG	0 to 1	100%	85%	60%
	2 or greater	0%	15%	16%
	unknown	–	–	24%
Primary tumor site	Oral cavity	27%	22%	31%
	Oropharynx	38%	44%	33%
	Nasopharynx	–	7%	–
	Larynx	25%	11%	15%
	Cutaneous	–	10%	4%
	Hypopharynx	13%	–	4%
	Other/Unknown	–	6%	14%
Staging at diagnosis	Stage I, II	–	13%	18%
	Stage III, IV	–	87%	71%
	Unknown	–	–	11%
HPV status	p16+	21%	40%	27%
	p16-/not tested	79%	60%	73%

### Biomarker development

These 103 samples served as the training set for the development of the biomarker. RNA-seq data were analyzed with a proprietary analysis pipeline to integrate gene expression signatures associated with eight immune cell types (Schillebeeckx et al. 2020) and five additional T cell states (Schillebeeckx et al. 2022), as well as other immune related gene expression data in each sample (Fig. 1A). Patients were grouped according to disease control status as determined by clinical assessment at 8–10 weeks (Eisenhauer et al. 2009). Non-progressors were patients with complete response (CR), partial response (PR), or stable disease (SD). Progressors were patients with progressive disease (PD). Median progression free survival (PFS) for CR, PR, SD, and PD was undetermined, 8.4, 6.8, and 1.8 months, respectively (Fig. 1B). The gene expression data and disease control label served as input to a machine-learning based approach we term Predictive Immune Modeling to generate a multidimensional biomarker that predicts disease control for anti-PD-1 monotherapy in RM-HNSCC. Sixty-two candidate feature genes were pre-selected (Fig. 1C and Table S1; Schillebeeckx et al. 2020, 2022) to minimize the risk of over-fitting, and a forward-feature selection method was used to select the features in the final model (see methods).

### Performance of biomarker

To minimize inaccuracies and biased performance associated with the more traditional approach of segregating a single training and a single validation cohort in a patient pool of 103 samples, bootstrapping cross validation was used to best estimate future performance (Efron 1979). By maximizing the size of the training set, we better capture the diversity of the patient population. Considering *n* unique patient samples, patients are chosen *n* times with replacement and used for training a model. Using this trained model, a score (“out-of-bag score”) is generated for the remainder of patients. This process was done 1000 times, and the out-of-bag score of each patient was averaged to generate the final OncoPrism Score. The OncoPrism Score, a value from 0 to 100, indicates the likelihood of non-progression with anti-PD-1 monotherapy treatment.

**Fig. 2:**
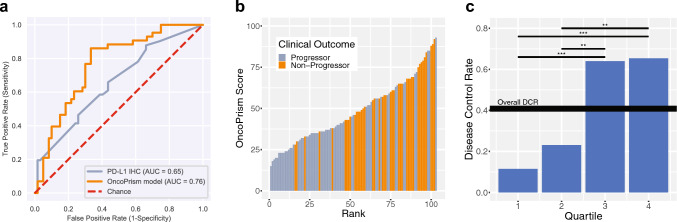
Higher OncoPrism Scores are associated with disease control with anti-PD-1 therapy **A** ROC curve showing the performance of OncoPrism-HNSCC compared to PD-L1 IHC across all thresholds. An out-of-bag (OOB) method was used to assess performance. OncoPrism-HNSCC (orange) has an area under the curve (AUC) of 0.76, while PD-L1 IHC (grey) has an AUC of 0.65. The dashed line (red) is performance equivalent to chance. **B** Patients were ranked along the x-axis according to their OncoPrism Score (y-axis) and colored according to their actual clinical outcome (progressors = grey, non-progressors = orange) **C** Samples were ranked according to their OncoPrism Score and divided into quartiles for assessment. The disease control rate (DCR) for all patients is represented by the solid line. The blue bars represent the actual DCR for each quartile not a mean. Significant differences among quartiles were determined using Fisher’s Exact test (*p < 0.05, **p < 0.01, ***p < 0.001)

A receiver operating characteristic (ROC) curve was generated to assess the performance of the biomarker across all thresholds (Fig. 2A, orange line). The area under the curve (AUC) was 0.76. An AUC of 1 represents perfect performance while an AUC of 0.5 represents performance equal to random chance. In contrast, the traditional, single analyte on-label diagnostic, PD-L1 IHC, had an AUC of 0.65 (n = 100; Fig. 2A, gray line). Next we looked at the correlation between patient OncoPrism Score and disease control (Fig. 2B). The likelihood of non-progression increased as the OncoPrism Score increased. This correlation between OncoPrism Score and disease control demonstrates the power of the biomarker to predict disease control with anti-PD-1 monotherapy in a continuous manner. To quantitate this relationship, we divided OncoPrism Scores into quartiles and measured the Disease Control Rate (DCR) in each quartile (Fig. 2C). The overall DCR for all quartiles was 41%. DCR was 12% in the lowest quartile (OncoPrism Scores 0–35), 23% in the second quartile (OncoPrism Scores 36–46), 64% in the third quartile (OncoPrism Scores 47–62), and 65% in the highest quartile (OncoPrism Scores 63–100). Relative to the DCR for all patients, non-progressors were significantly underrepresented in the first quartile and significantly enriched in the third and fourth quartiles (Fisher’s exact test, p < 0.01).

### Development of the OncoPrism-HNSCC test utilizing biomarker

RM-HNSCC patients are commonly treated with anti-PD-1 plus platinum-based chemotherapy (combination therapy) by default (Le et al. 2020). We sought to develop a test to identify patients who will benefit from anti-PD-1 monotherapy. The biomarker and OncoPrism Score described above provide the foundation for OncoPrism-HNSCC, a test to guide this treatment decision. Because the upper two quartiles of patients had significantly higher DCR than the lower two quartiles (p < 0.01; Fig. 2C), we used the median score (46) as the threshold for predicting disease control. The first and second quartiles combined constituted predicted progressors, while the third and fourth quartiles combined represented predicted non-progressors. This median threshold balances the predictive power of the test with the size of the patient population for whom the test would improve outcomes relative to existing biomarkers and standards of care.

**Fig. 3:**
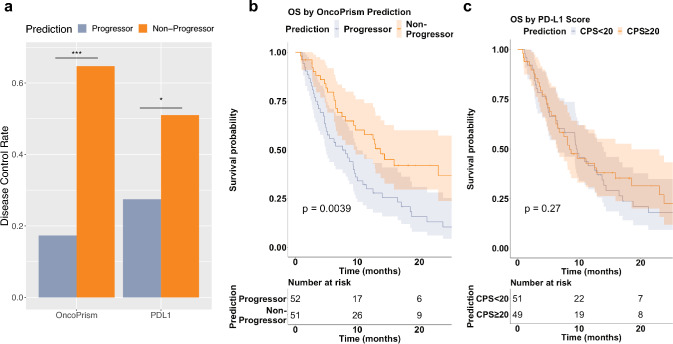
OncoPrism-HNSCC predicts disease control and overall survival (OS) better than PD-L1 IHC. **A** DCR is significantly higher for OncoPrism-HNSCC predicted non- progressors than for OncoPrism-HNSCC predicted progressors (p < 0.001). DCR for PD- L1 CPS ≥ 20 (“non-progressor”) is significantly higher than CPS < 20 (“progressor”; p = 0.02). Bars represent the actual DCRs not a mean. Significant differences among quartiles were determined using Fisher’s Exact test. **B** OncoPrism-HNSCC predicted non-progressors have significantly longer OS than predicted progressors (p = 0.004; n = 103). OS is measured from the time of first anti-PD-1 treatment. **C** PD-L1 IHC CPS ≥ 20 patients (corresponding to a PD-L1 predicted non-progressor) do not have longer OS than CPS < 20 (p = 0.7; n = 100). OS is measured from the time of first anti-PD-1 treatment

Using this categorical labeling, the DCR for predicted non-progressors was 65%, significantly higher than the DCR of 17% for predicted progressors (Fisher’s exact test, p < 0.001; Fig. 3A). The relative risk of progression for predicted progressors relative to non-progressors was 2.34 (p < 0.001). Although we designed the model to predict disease control, Overall Response Rate (ORR) was also significantly higher in predicted non-progressors (41%) compared to predicted progressors (13%; p = 0.002; Fig. S1). Importantly, predicted non-progressors also had significantly longer overall survival (OS; median = 13.7 months) than predicted progressors (median = 7.9 months; Fig. 3B; p = 0.004).

### OncoPrism-HNSCC is more predictive than PD-L1 IHC

OncoPrism-HNSCC performed well compared to the existing anti-PD-1 CDx, PD-L1 IHC. PD-L1 IHC and quantitation was performed on 100 samples and used to evaluate PD-L1 IHC performance. Pembrolizumab is approved as a first-line therapy for patients with a PD-L1 IHC combined positive score greater than or equal to one (CPS ≥ 1). Samples with a PD-L1 IHC CPS ≥ 1 had lower DCR (44%) than OncoPrism-HNSCC predicted non-progressors (65%; p = 0.02; compare Table S3 to Fig. 3A). In practice, CPS ≥ 20 is a more common threshold than CPS > 1 for monotherapy treatment decisions (Pfister et al. 2023). Even with this higher PD-L1 threshold (CPS ≥ 20), the DCR for PD-L1 IHC increases only marginally to 51%, compared to 27% for CPS < 20 (p = 0.02; Table S3).

Additionally, OncoPrism-HNSCC outperformed PD-L1 CPS ≥ 20 across multiple metrics, including higher sensitivity (0.79 vs 0.64, p = 0.005) and specificity (0.70 vs 0.61, p = 0.009; Table 2). While the OncoPrism-HNSCC and PD-L1 predictions were concordant for most samples (73/100), for samples where the tests disagreed, PD-L1 IHC had more false positives (12) and false negatives (7) than OncoPrism-HNSCC (6 and 2, respectively; Table S4).

**Table 2:** Performance metrics

	OncoPrism	PD-L1	Ratio^a^
Accuracy	0.74	0.62	1.19
Sensitivity	0.79	0.64	1.23
Specificity	0.7	0.61	1.16
Prevalence	0.41	0.39	1.05
FPR	0.3	0.39	0.75
FNR	0.21	0.36	0.6
PPV	0.65	0.51	1.27
NPV	0.83	0.73	1.14
LR+	2.66	1.63	1.63
LR−	0.3	0.59	0.51
DOR	8.76	2.75	3.18

*FPR* false positive rate, *FNR* false negative rate, *PPV* positive predictive value, *NPV* negative predictive value, *LR+* positive likelihood ratio, *LR–* negative likelihood ratio, *DOR* diagnostic odds ratio

^a^Ratio of OncoPrism to PD-L1

**Fig. 4:**
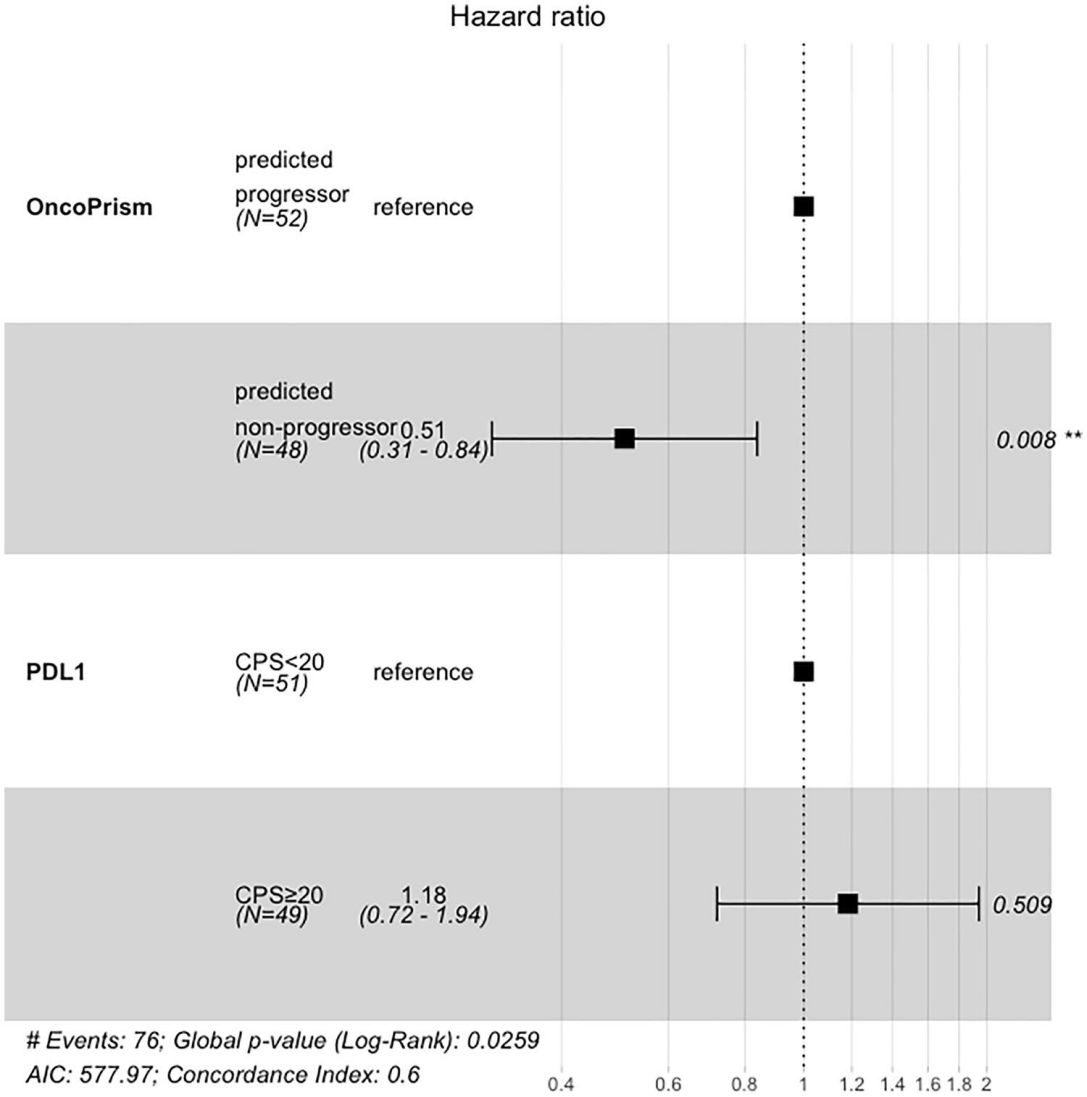
Cox proportional hazards model of overall survival (OS). Patients with complete OncoPrism-HNSCC and PD-L1 data were included (n = 100). OncoPrism-HNSCC predicted non-progressors have a Hazard Ratio of 0.51 relative to predicted progressors (p = 0.008). There is no significant difference in OS between PD-L1 < 20 and PD-L1 ≥ 20

Unlike with OncoPrism-HNSCC, patients with PD-L1 CPS ≥ 20 did not have longer OS (Fig. 3C). We used the Cox proportional hazards model to assess the relationships between OncoPrism-HNSCC prediction, PD-L1 IHC CPS category, and survival in the 100 samples with OncoPrism-HNSCC, PD-L1, and survival data (Fig. 4). Relative to OncoPrism-HNSCC predicted progressors, predicted non-progressors had a hazard ratio (HR) of 0.51 (p < 0.01), indicating a significant positive association with survival. For PD-L1 IHC, relative to CPS < 20 the HR for CPS ≥ 20 was 1.18 (p = 0.51), indicating no association with survival.

### OncoPrism-HNSCC performance is similar across RM-HNSCC subtypes

Although the study was not powered to statistically evaluate sub-groups, OncoPrism- HNSCC performance was qualitatively similar across clinical variables including primary tumor site and HPV status. While the overall DCR varied across primary tumor sites, in each site the predicted non-progressors had a higher DCR than predicted progressors (Table S5). Likewise, while the DCR in HPV-positive (p16 +) patients was higher than in HPV-negative (p16-) patients, as expected (Weinberger et al. 2006; Singhi and Westra 2010; Cai et al. 2014; Pfister et al. 2023), OncoPrism-HNSCC performance was similar regardless of p16 status (Table S6). Additionally, we used Cox proportional hazards model to explore clinical factors associated with OS (Fig. S2). OncoPrism-HNSCC predicted non-progressors were positively associated with survival. Eastern Cooperative Oncology Group (ECOG) score of 2 and a positive or unknown p16 status were negatively associated with survival. PD-L1 status, overall stage, smoker status, and primary tumor site were not correlated with survival.

## Discussion

OncoPrism-HNSCC predicts disease control and OS in response to anti-PD-1 monotherapy in a 103 patient dataset from 11 clinical sites using out-of-bag methods (Fig. 3A–B). In addition, although OncoPrism-HNSCC was trained to predict disease control, it also significantly predicts overall response (Fig. S1). While PD-L1 IHC was also moderately predictive of DCR, PD-L1 did not predict overall response or OS in our study population (Fig. 3A and C; Fig. S1). OncoPrism-HNSCC also had significantly higher sensitivity and specificity than PD-L1 IHC (Table 2).

Current biomarkers in RM-HNSCC are only moderately predictive, causing clinicians to treat more aggressively with combination therapy (anti-PD-1 therapy combined with platinum-chemotherapy) than would be required with better biomarkers (Chow 2020; Le et al. 2020). In KEYNOTE-048, patients with PD-L1 IHC CPS ≥ 20 had only moderately higher disease control with pembrolizumab monotherapy than all monotherapy patients regardless of PD-L1 status (DCR of 53% vs 44%, respectively; Burtness et al. 2019). Likewise in our study, the 51% DCR in monotherapy-treated patients with CPS ≥ 20 was only moderately better than the 39% DCR in the total monotherapy-treated patient population (Table S3). Using the FDA approved monotherapy threshold of CPS ≥ 1 reduced the predictive power of PD-L1 IHC even more. Importantly, in KEYNOTE-048, the 64% DCR in all combination therapy patients was higher than the DCR to anti-PD-1 monotherapy even in patients with CPS ≥ 20 (DCR = 53%). Because the DCR for combination-treated patients (regardless of PD-L1 status) is higher than monotherapy-treated patients even with high PD-L1, many clinicians default to the more aggressive combination therapy. However, treatment with chemotherapy—with or without anti-PD-1—causes significant patient quality of life issues, increased serious adverse events, and higher healthcare costs (Ferris et al. 2016; Harrington et al. 2017; Brockstein and Vokes 2023).

With any biomarker used to make patient treatment decisions, it is important to avoid withholding an effective treatment from patients who would benefit. OncoPrism-HNSCC has a higher sensitivity than PD-L1 IHC (0.79 vs. 0.64, p = 0.005; Table 2), corresponding to a lower number of false negatives wherein the test incorrectly predicts progression. In this study PD-L1 IHC had 14 false negatives compared to just 9 for OncoPrism-HNSCC (Table S4). Seven of these false negatives were common to both tests, with just two false negatives unique to OncoPrism-HNSCC. In addition, seven patients who are PD-L1 IHC false negatives were correctly predicted as non-progressors by OncoPrism-HNSCC. While there remain individual patients who would not receive the optimal therapy with OncoPrism-HNSCC, there are fewer such patients when following the OncoPrism-HNSCC prediction compared to the existing PD-L1 IHC prediction. Future work will focus on further reducing false negatives.

OncoPrism-HNSCC predicts with high specificity patients who will have disease control in response to monotherapy, potentially enabling clinicians to avoid overtreatment in a subset of patients. In our dataset, monotherapy-treated OncoPrism-HNSCC-predicted non-progressors had a DCR of 65% compared to just 51% for CPS ≥ 20 (Fig. 3A). Future studies should compare monotherapy and combination therapy outcomes among predicted progressors and non-progressors. In contrast to OncoPrism-HNSCC predictions, PD-L1 expression was only moderately correlated with monotherapy DCR in our study (Fig. 3A and Table S3), and not at all correlated with OS (Fig. 3C). Further, the data from this study indicate that relative to CPS ≥ 20, OncoPrism-HNSCC provides superior sensitivity (0.79 vs 0.64, p = 0.005) and specificity (0.70 vs 0.61, p = 0.009) in monotherapy patients relative to PD-L1 IHC (Table 2). While these results are limited by the lack of an independent validation set, additional studies are ongoing to validate these results in additional independent cohorts of patients.

## Conclusions

OncoPrism-HNSCC is a novel multidimensional RNA-seq biomarker-based clinical test that predicts DCR, ORR, and OS in monotherapy anti-PD-1-treated RM-HNSCC patients. OncoPrism-HNSCC fills a clinical need in the treatment of HNSCC by identifying with high sensitivity and specificity patients who may benefit from monotherapy. This ability to better predict patient disease control to anti-PD-1 monotherapy provides an opportunity to give patients the most effective treatment option and avoid unnecessary chemotherapy.

### Supplementary Information

Below is the link to the electronic supplementary material.

Supplementary file1 (EPS 8 KB) Overall Response Rate (ORR) is significantly higher for OncoPrism-HNSCC predicted non-progressors than for OncoPrism-HNSCC predicted progressors (p=0.002). ORR is not significantly different between PD-L1 CPS≥20 (“non-progressor”) and CPS<20 (“progressor”)

Supplementary file2 (TIFF 2200 KB) Cox Proportional Hazards Model of overall survival with potential prognostic factors. Patients with complete OncoPrism-HNSCC, PD-L1 and clinical data were included (n=100). The only significant prognostic factors were label, ECOG score, and p16 status

Supplementary file3 (PDF 45 KB)

Supplementary file4 (PDF 12 KB)

Supplementary file5 (PDF 12 KB)

Supplementary file6 (PDF 12 KB)

Supplementary file7 (PDF 9 KB)

Supplementary file8 (PDF 9 KB)

## Data Availability

The datasets used and/or analyzed during the current study are available from the corresponding author upon reasonable request.

## Abbreviations

RM-HNSCC: Recurrent/metastatic-head and neck squamous cell carcinoma
IHC: Immunohistochemistry
OS: Overall survival
HNC: Head and neck cancer
CDx: Companion diagnostic
PPV: Positive predictive value
NPV: Negative predictive value
CPS: Combined positive score
DCR: Disease control rate
FFPE: Formalin-fixed, paraffin-embedded
PREDAPT: Predicting immunotherapy efficacy from analysis of pre-treatment tumor biopsies
CR: Complete response
PR: Partial response
SD: Stable disease
PD: Progressive disease
ORR: Overall response rate
ICI: Immune checkpoint inhibitor
ROC: Receiver operating characteristic
AUC: Area under the curve
HR: Hazard ratio
HPV: Human papillomavirus
ECOG: Eastern cooperative oncology group

## References

[CR1] Argiris A, Karamouzis MV, Raben D, Ferris RL (2008). Head and neck cancer. Lancet.

[CR2] Brockstein BE, Vokes EE (2023) Treatment of metastatic and recurrent head and neck cancer. In Post TW, Posner MR, and Shah S (Eds.), *UpToDate*, Waltham, MA. Available from https://www.uptodate.com/contents/treatment-of-metastatic-and-recurrent-head-and-neck-cancer/print. Accessed 12 May 2023

[CR3] Burtness B, Harrington KJ, Greil R (2019). Pembrolizumab alone or with chemotherapy versus cetuximab with chemotherapy for recurrent or metastatic squamous cell carcinoma of the head and neck (KEYNOTE-048): a randomised, open-label, phase 3 study. Lancet.

[CR4] Cai C, Chernock RD, Pittman ME (2014). Keratinizing-type squamous cell carcinoma of the oropharynx: p16 overexpression is associated with positive high-risk HPV status and improved survival. Am J Surg Pathol.

[CR5] Chow LQM (2020). Head and neck cancer. N Engl J Med.

[CR6] Cohen EEW, Bryan Bell R, Bifulco CB (2019). The society for immunotherapy of cancer consensus statement on immunotherapy for the treatment of squamous cell carcinoma of the head and neck (HNSCC). J Immunother Cancer.

[CR7] Cohen EEW, Soulières D, Le Tourneau C (2019). Pembrolizumab versus methotrexate, docetaxel, or cetuximab for recurrent or metastatic head-and-neck squamous cell carcinoma (KEYNOTE-040): a randomised, open-label, phase 3 study. Lancet.

[CR8] Efron B (1979). Bootstrap methods: another look at the jackknife. Ann Stat.

[CR9] Eisenhauer EA, Therasse P, Bogaerts J (2009). New response evaluation criteria in solid tumours: revised RECIST guideline (version 1.1). Eur J Cancer.

[CR10] Ferris RL, Blumenschein G, Fayette J (2016). Nivolumab for recurrent squamous-cell carcinoma of the head and neck. N Engl J Med.

[CR11] Gu Z, Gu L, Eils R (2014). Circlize implements and enhances circular visualization in R. Bioinformatics.

[CR12] Gu Z, Eils R, Schlesner M (2016). Complex heatmaps reveal patterns and correlations in multidimensional genomic data. Bioinformatics.

[CR13] Hanna GJ, Lizotte P, Cavanaugh M (2018). Frameshift events predict anti-PD-1/L1 response in head and neck cancer. JCI Insight.

[CR14] Harrington KJ, Ferris RL, Blumenschein G (2017). Nivolumab versus standard, single-agent therapy of investigator’s choice in recurrent or metastatic squamous cell carcinoma of the head and neck (CheckMate 141): health-related quality-of-life results from a randomised, phase 3 trial. Lancet Oncol.

[CR15] LaFranzo NA, Flanagan KC, Quintanilha D (2020) Predictive immune modeling of solid tumors. J Vis Exp. 10.3791/60645 10.3791/60645 32176206

[CR16] Larkins E, Blumenthal GM, Yuan W (2017). FDA approval summary: pembrolizumab for the treatment of recurrent or metastatic head and neck squamous cell carcinoma with disease progression on or after platinum-containing chemotherapy. Oncologist.

[CR17] Le X, Ferrarotto R, Wise-Draper T, Gillison M (2020). Evolving role of immunotherapy in recurrent metastatic head and neck cancer. JNCCN.

[CR18] Lu S, Stein JE, Rimm DL (2019). Comparison of biomarker modalities for predicting response to PD-1/PD-L1 checkpoint blockade: a systematic review and meta-analysis. JAMA Oncol.

[CR19] Muro K, Chung HC, Shankaran V (2016). Pembrolizumab for patients with PD-L1-positive advanced gastric cancer (KEYNOTE-012): a multicentre, open-label, phase 1b trial. Lancet Oncol.

[CR20] Pfister DG, Spencer S, Adkins D, et al (2023) National Comprehensive Cancer Network. Head and Neck Cancers Version 2.2023. https://www.nccn.org/professionals/physician_gls/pdf/head-and-neck.pdf. Accessed 19 May 2023

[CR21] Pulte D, Brenner H (2010). Changes in Survival in Head and Neck Cancers in the Late 20th and Early 21st Century: A Period Analysis. Oncologist.

[CR22] R Core Team (2021) R: a language and environment for statistical computing. https://www.R-project.org/. Accessed 9 Mar 2023

[CR23] Sacco AG, Cohen EE (2015). Current treatment options for recurrent or metastatic head and neck squamous cell carcinoma. J Clin Oncol.

[CR24] Schillebeeckx I, Armstrong JR, Forys JT (2020). Analytical performance of an immunoprofiling assay based on RNA models. J Mol Diagn.

[CR25] Schillebeeckx I, Earls J, Flanagan KC (2022). T cell subtype profiling measures exhaustion and predicts anti-PD-1 response. Sci Rep.

[CR26] Singhi AD, Westra WH (2010). Comparison of human papillomavirus in situ hybridization and p16 immunohistochemistry in the detection of human papillomavirus-associated head and neck cancer based on a prospective clinical experience. Cancer.

[CR27] Therneau TM (2023) Survival Analysis [R package survival version 3.5-5]

[CR28] Therneau TM, Grambsch PM (2000). Modeling survival data: extending the cox model.

[CR29] Vermorken JB, Mesia R, Rivera F (2008). Platinum-based chemotherapy plus cetuximab in head and neck cancer. N Engl J Med.

[CR30] Weinberger PM, Yu Z, Haffty BG (2006). Molecular classification identifies a subset of human papillomavirus- associated oropharyngeal cancers with favorable prognosis. J Clin Oncol.

