# Supplementary figures and images for: Multidimensional biomarker predicts disease control in response to immunotherapy in recurrent or metastatic head and neck squamous-cell carcinoma
Source: J Cancer Res Clin Oncol. 2023 Aug 8;149(15):14125–36. doi: 10.1007/s00432-023-05205-z (PMC10590294; doi:10.1007/s00432-023-05205-z)

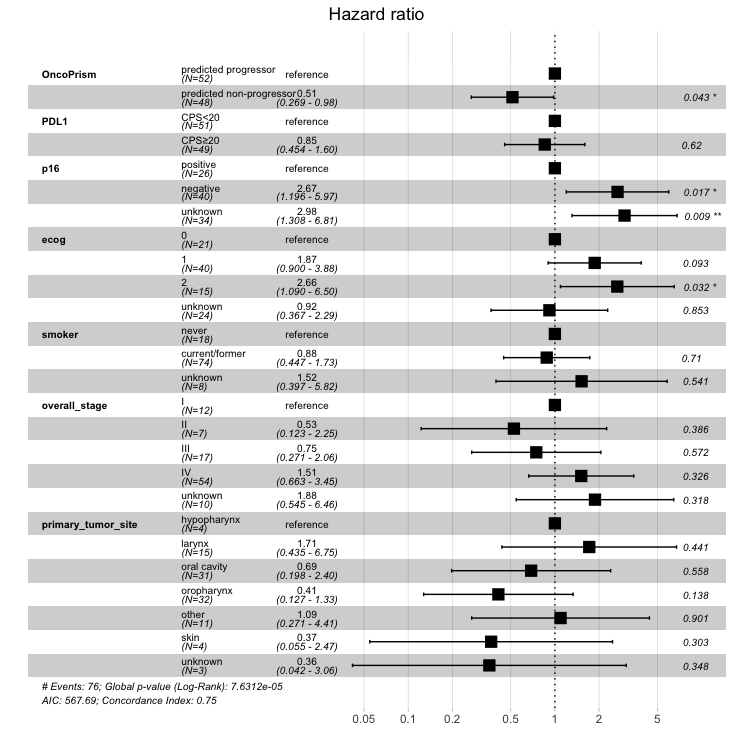

Supplement: Supplementary file 2 — Supplementary file2 (TIFF 2200 KB) Cox Proportional Hazards Model of overall survival with potential prognostic factors. Patients with complete OncoPrism-HNSCC, PD-L1 and clinical data were included (n=100). The only significant prognostic factors were label, ECOG score, and p16 status [file 432_2023_5205_MOESM2_ESM.tiff]
