# Supplementary material for: Multidimensional biomarker predicts disease control in response to immunotherapy in recurrent or metastatic head and neck squamous-cell carcinoma
Source: J Cancer Res Clin Oncol. 2023 Aug 8;149(15):14125–36. doi: 10.1007/s00432-023-05205-z (PMC10590294; doi:10.1007/s00432-023-05205-z)
Supplement: Supplementary file 3 — Supplementary file3 (PDF 45 KB) [file 432_2023_5205_MOESM3_ESM.pdf]

Table S1: Candidate Features Used for Forward Feature Selection

Feature	Type
CD4 T Cell	Composite
CD8 T Cell	Composite
CD19 B Cell	Composite
CD14 Monocyte	Composite
CD56 NK Cell	Composite
M1 Macrophage	Composite
M2 Macrophage	Composite
Treg T Cell	Composite
EM T Cell	Composite
Exhausted T Cell	Composite
Naive T Cell	Composite
Activated T Cell	Composite
CM T Cell	Composite
sum Immune Cells	Composite
sum T Cells	Composite
CD8A	Gene Expression
LAG3	Gene Expression
HAVCR2	Gene Expression
CD244	Gene Expression
PDCD1LG2	Gene Expression
CD80	Gene Expression
ICOS	Gene Expression
IDO1	Gene Expression
CD274	Gene Expression
CD48	Gene Expression
PDCD1	Gene Expression
CD96	Gene Expression
BTLA	Gene Expression
TNFSF4	Gene Expression
TNFSF18	Gene Expression
CTLA4	Gene Expression
CD276	Gene Expression
CD40	Gene Expression
LGALS9	Gene Expression
PVRL2	Gene Expression
CD70	Gene Expression
CD47	Gene Expression
ARG1	Gene Expression
CD28	Gene Expression

TNFRSF18	Gene Expression
CD27	Gene Expression
CD40LG	Gene Expression
TMIGD2	Gene Expression
TNFRSF4	Gene Expression
TNFSF15	Gene Expression
TNFRSF25	Gene Expression
HHLA2	Gene Expression
STAT1	Gene Expression
PRF1	Gene Expression
GZMA	Gene Expression
GZMB	Gene Expression
GZMH	Gene Expression
GZMK	Gene Expression
GZMM	Gene Expression
HLA-DRA	Gene Expression
HLA-B	Gene Expression
HLA-A	Gene Expression
HLA-C	Gene Expression
TAP1	Gene Expression
TAP2	Gene Expression
CXCL9	Gene Expression
CXCL13	Gene Expression
