# Supplementary material for: Multidimensional biomarker predicts disease control in response to immunotherapy in recurrent or metastatic head and neck squamous-cell carcinoma
Source: J Cancer Res Clin Oncol. 2023 Aug 8;149(15):14125–36. doi: 10.1007/s00432-023-05205-z (PMC10590294; doi:10.1007/s00432-023-05205-z)
Supplement: Supplementary file 4 — Supplementary file4 (PDF 12 KB) [file 432_2023_5205_MOESM4_ESM.pdf]

Table S2: Reasons for Failure

None (Included in study)	103
Unacceptable Treatment Regimen	4
Excessive Time to Treatment	12
Unacceptable Specimen Site	13
Incomplete Data	1
Tumor Cellularity <10%	22
Fail RNA QC	9
Fail Library QC	1
Fail Analytical QC	7
**Total**	**172**
