# Supplementary material for: Multidimensional biomarker predicts disease control in response to immunotherapy in recurrent or metastatic head and neck squamous-cell carcinoma
Source: J Cancer Res Clin Oncol. 2023 Aug 8;149(15):14125–36. doi: 10.1007/s00432-023-05205-z (PMC10590294; doi:10.1007/s00432-023-05205-z)
Supplement: Supplementary file 5 — Supplementary file5 (PDF 12 KB) [file 432_2023_5205_MOESM5_ESM.pdf]

Table S3: DCR by PD-L1 Combined Positive Score

	progressors	non-progressors	total	DCR
CPS<1	17	5	22	23%
CPS≥1	44	34	78	44%
CPS=1-19	20	9	29	31%
CPS<20	37	14	51	27%
CPS≥20	24	25	49	51%
All	61	39	100	39%

Note:

DCR for CPS≥20 is significantly higher than both CPS<1 and CPS<20 (Fisher's Exact test, p=0.04 and p=0.02, respectively. No other comparisons were significant.
