# Supplementary material for: Multidimensional biomarker predicts disease control in response to immunotherapy in recurrent or metastatic head and neck squamous-cell carcinoma
Source: J Cancer Res Clin Oncol. 2023 Aug 8;149(15):14125–36. doi: 10.1007/s00432-023-05205-z (PMC10590294; doi:10.1007/s00432-023-05205-z)
Supplement: Supplementary file 6 — Supplementary file6 (PDF 12 KB) [file 432_2023_5205_MOESM6_ESM.pdf]

Table S4: Comparison of OncoPrism and PDL1 IHC

	PD-L1 TP	PD-L1 FN	PD-L1 TN	PD-L1 FP
OncoPrism TP	23	7	-	-
OncoPrism FN	2	7	-	-
OncoPrism TN	-	-	31	12
OncoPrism FP	-	-	6	12

Abbreviations:

TP, True Positive, correctly predicted non-progressors;

FN, False Negative, non-progressors incorrectly predicted as progressors;

TN, True Negative, correctly predicted progressors;

FP, False Positive, progressors incorrectly predicted as non-progressors
