# Supplementary material for: Multidimensional biomarker predicts disease control in response to immunotherapy in recurrent or metastatic head and neck squamous-cell carcinoma
Source: J Cancer Res Clin Oncol. 2023 Aug 8;149(15):14125–36. doi: 10.1007/s00432-023-05205-z (PMC10590294; doi:10.1007/s00432-023-05205-z)
Supplement: Supplementary file 7 — Supplementary file7 (PDF 9 KB) [file 432_2023_5205_MOESM7_ESM.pdf]

Table S5: DCR by Primary Tumor Site

OncoPrism Prediction	Oropharynx	Oral Cavity	Larynx	Other/Unknown	Overall
progressor	33%	17%	0%	10%	17%
non-progressor	84%	64%	33%	50%	65%
all	62%	38%	13%	32%	41%
# of patients	34	32	15	22	103
