# Supplementary material for: Multidimensional biomarker predicts disease control in response to immunotherapy in recurrent or metastatic head and neck squamous-cell carcinoma
Source: J Cancer Res Clin Oncol. 2023 Aug 8;149(15):14125–36. doi: 10.1007/s00432-023-05205-z (PMC10590294; doi:10.1007/s00432-023-05205-z)
Supplement: Supplementary file 8 — Supplementary file8 (PDF 9 KB) [file 432_2023_5205_MOESM8_ESM.pdf]

Table S6: DCR by HPV Status

OncoPrism Prediction	p16-negative	p16-positive	unknown	Overall
progressor	26%	27%	15%	17%
non-progressor	44%	82%	57%	65%
all	34%	61%	32%	41%
# of patients	41	28	34	103
